# Timing and source of subtype-C HIV-1 superinfection in the newly infected partner of Zambian couples with disparate viruses

**DOI:** 10.1186/1742-4690-9-22

**Published:** 2012-03-20

**Authors:** Colleen S Kraft, Debby Basu, Paulina A Hawkins, Peter T Hraber, Elwyn Chomba, Joseph Mulenga, William Kilembe, Naw H Khu, Cynthia A Derdeyn, Susan A Allen, Olivier Manigart, Eric Hunter

**Affiliations:** 1 Emory Vaccine Center at Yerkes National Primate Research Center, Emory University, Atlanta, GA, USA; 2 Los Alamos National Laboratory, Los Alamos, NM, USA; 3 Zambia Emory HIV Research Project, ZEHRP, Lusaka, Zambia; 4 Department of Global Health, Rollins School of Public Health, Emory University, Atlanta, GA, USA; 5 Projet San Francisco, Rwanda Zambia HIV Research Group, RZHRG, Kigali, Rwanda

## Abstract

**Background:**

HIV-1 superinfection occurs at varying frequencies in different at risk populations. Though seroincidence is decreased, in the negative partner of HIV-discordant couples after joint testing and counseling in the Zambia Emory HIV Research Project (ZEHRP) cohort, the annual infection rate remains relatively high at 7-8%. Based on sequencing within the gp41 region of each partner's virus, 24% of new infections between 2004 and 2008 were the result of transmission from a non-spousal partner. Since these seroconvertors and their spouses have disparate epidemiologically-unlinked viruses, there is a risk of superinfection within the marriage. We have, therefore, investigated the incidence and viral origin of superinfection in these couples.

**Results:**

Superinfection was detected by heteroduplex mobility assay (HMA), degenerate base counting of the gp41 sequence, or by phylogenetic analysis of the longitudinal sequences. It was confirmed by full-length *env *single genome amplification and phylogenetic analysis. In 22 couples (44 individuals), followed for up to five years, three of the newly infected (initially HIV uninfected) partners became superinfected. In each case superinfection occurred during the first 12 months following initial infection of the negative partner, and in each case the superinfecting virus was derived from a non-spousal partner. In addition, one probable case of intra-couple HIV-1 superinfection was observed in a chronically infected partner at the time of his seroconverting spouse's initial viremia. Extensive recombination within the *env *gene was observed following superinfection.

**Conclusions:**

In this subtype-C discordant couple cohort, superinfection, during the first year after HIV-1 infection of the previously negative partner, occurred at a rate similar to primary infection (13.6% [95% CI 5.2-34.8] vs 7.8% [7.1-8.6]). While limited intra-couple superinfection may in part reflect continued condom usage within couples, this and our lack of detecting newly superinfected individuals after one year of primary infection raise the possibility that immunological resistance to intra-subtype superinfection may develop over time in subtype C infected individuals.

## Background

HIV-1 superinfection presents an additional concern to the already challenging problem of HIV-1 vaccine design in the face of the virus's rapid evolution [1]. Superinfection is defined as a reinfection by a heterologous HIV-1 strain after a primary immune response has already been mounted [2]. Superinfection and coinfection (primary infection with two genetically distinct viruses) differ based on whether the second infection is contracted prior to or after the host immune response has been mounted [3]. The first documented case of superinfection was identified in a high-risk MSM individual, initially infected with a CRF01_AE subtype followed by a subtype B superinfection after two years [4]. Several other cases have been reported, demonstrating a spectrum of intersubtype [5-12], intergroup [13] and intrasubtype [14-17] superinfections.

Many studies have raised questions about the frequency of superinfection and were unable to identify HIV-1 superinfection in the populations under investigation [18-21]. Despite these doubts, HIV-1 superinfection has now been seen to occur at frequencies comparable to primary infection in certain cohorts [5,17]. The behavioral aspects of these cohorts impact transmission [22] and the interplay between the risk for re-exposure [23], as well as the regional HIV-1 prevalence have been thought to influence the likelihood of HIV-1 superinfection in a given population [3].

It is known that HIV-1 superinfection occurs despite broad CD8+ T-cell [14] and cross-reacting neutralizing antibody responses [24], although it appears that there is less likelihood for HIV-1 superinfection later in the course of HIV infection [17,25,26]. Studies have evaluated the neutralizing antibody population around the time of superinfection and demonstrated both lack of neutralizing antibody [27] as well as robust neutralizing responses [24].

HIV-1 superinfection has clinical ramifications. Transmission of drug resistant variants through superinfection has been well described [16,17,28-30] and there has been evidence of increased viral load set-points in individuals who are dually infected [31,32] or superinfected [14,16]. The numerous circulating recombinant forms of HIV-1 demonstrate that dual infection of individuals [9,33,34] and the resulting superinfection can contribute to the overall diversity of a virus population. Modeling has shown that intrasubtype superinfection may be as high as 15% in some populations based on evidence of recombination [35], and superinfection followed by recombination may contribute to immune escape within an individual [36].

Since 1994, the Zambia Emory HIV Research Project has followed a cohort of HIV-1 discordant couples, where one partner is HIV-infected and the other is HIV-uninfected. Joint counseling and condom provision in such couples can reduce transmission significantly [37-40]. When HIV-1 infections occur, approximately one in eight are acquired from non-spousal partners, leading to a couple infected with genetically distinct viruses [41]. We have followed 22 of these epidemiologically unlinked couples longitudinally for at least 1 year and up to 5 years, to determine the frequency and nature of superinfection in this cohabiting heterosexual population. We observed superinfection in four out of 44 individuals, but only one of these involved transmission of virus from one spouse to the other. The other superinfections resulted from transmissions from non-spousal partners within one year following a primary HIV-1 infection acquired in an extra-marital relationship. Thus, superinfection from non-spousal partners occurs more commonly than between spousal partners in this cohort, although evidence for continued condom use between spousal partners could limit the incidence of intra-couple HIV-1 superinfection.

## Results

### Selection of the study couples

Two hundred and two HIV-1 discordant couples who seroconverted to concordant infected status (both partners HIV-infected) from 2002-2008 in the ZEHRP cohort were screened for epidemiologic linkage as described previously [41]. In this subset of 202 couples, 49 (24%) were found to have partners with genetically distinct viruses (epidemiologically unlinked transmissions), and 22, selected as described in Methods, were screened for HIV-1 superinfection. Three approaches were employed (see Methods): 1) quantitation of degenerate bases in viral population sequences of the genomic regions encoding the ectodomain of gp41 and *gag*, 2) phylogenetic tree and Highlighter tool analyses of these sequences, and 3) heteroduplex mobility assay (HMA) of gp41 amplicons. If any of these methods suggested dual infection (either superinfection or co-infection - see Methods), longitudinal single genome amplification (SGA) of the full-length *env *gene was performed in order to further confirm and characterize the dual infection. Of the 22 acutely infected individuals, there were 9 women and 13 men; none of the participants reported undergoing antiretroviral therapy or engaging in risk behaviors other than heterosexual sex. The ZEHRP cohort is primarily (96.9%) subtype C [41,42], and as expected, all 44 of the individuals had primary infection with subtype C HIV-1. The length of screening for individuals ranged from 12-66 months, with at least 3 time points analyzed during the length of the screening for the acute partner.

### Identification of dual infections using PCR amplified gp41 sequences and highlighter tool analysis

**Figure 1:**
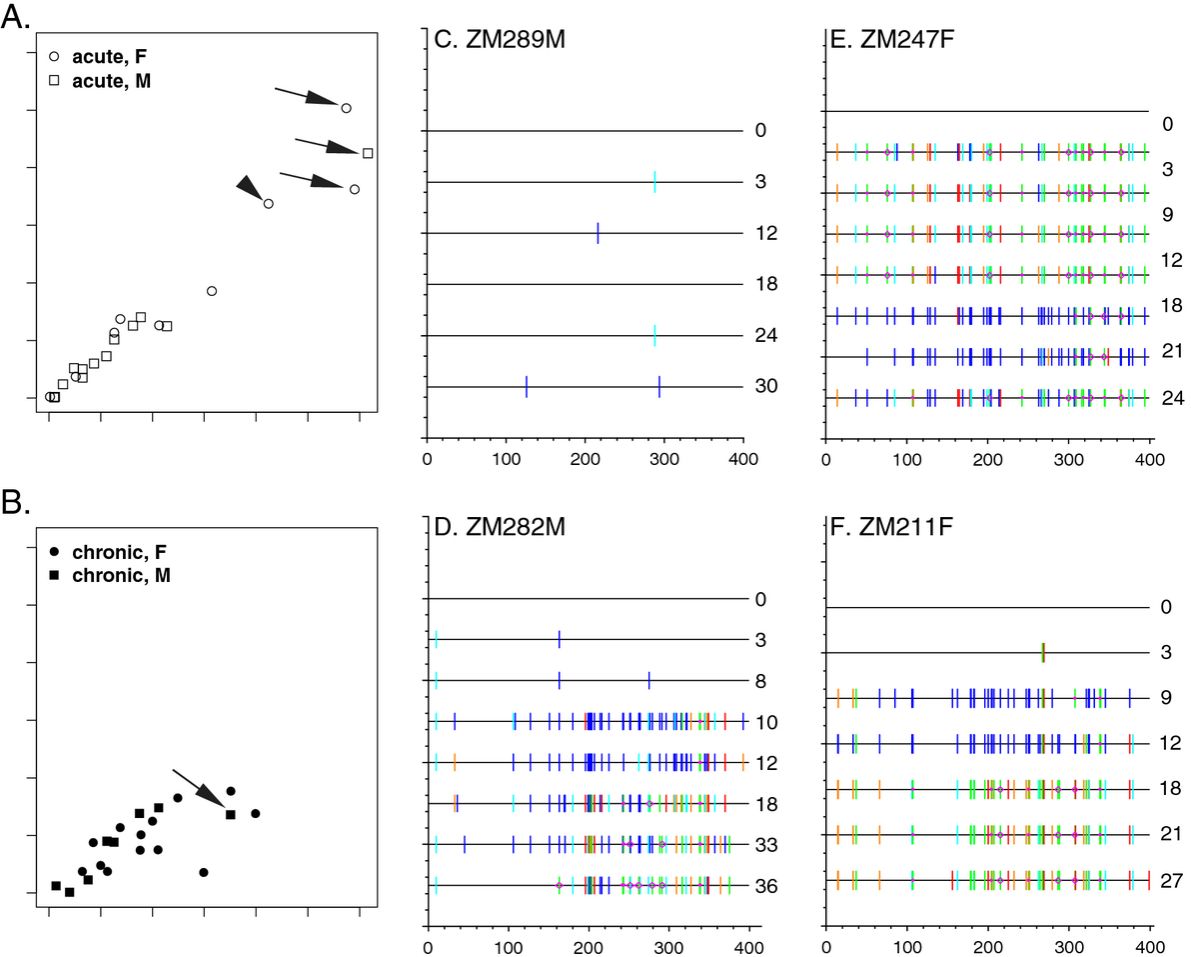
**Sequence and Highlighter Analysis of Longitudinal Samples Provides Evidence for Superinfection**. Comparison of maximum sequence divergence in gp41 versus the maximum number of degenerate bases at any time point within acutely infected individuals (**A**) and chronically infected individuals (**B**). The maximum percentage of degenerate bases is plotted on the y-axis; the maximum percentage of genetic distance is plotted along the x-axis. Black arrows indicate superinfected subjects; arrowhead indicates a subject co-infected with two variants from a single donor. Highlighter plots for gp41 sequences of ZM289M (**C**), ZM282M (**D**), ZM247F (**E**), and ZM211 F (**F**) sampled at 0 to 36 months post-seroconversion (shown on right of plot). The sequence at each time point is compared to the initial infecting HIV-1 gp41 sequence. Tick marks denote nucleotide changes from the seroconversion sequence (T = red, A = green, C = blue, G = yellow), with dark blue indicating degenerate bases (See Methods).

For each individual, a 399 bp fragment within the gp41 ectodomain region of the *env *gene was PCR amplified from each longitudinal sample time point as described in Methods. Degenerate bases (DB) were scored when a secondary peak exceeded 30% of the major peak height in the sequence traces. A comparison of the maximum number of degenerate bases at any time point (as a percentage of the 399-bp gp41 ectodomain sequenced) and the maximum pairwise distance (PD) between the month 0 virus sequence and that of the most divergent viral sequence was performed (Figure 1A, B). For the acutely infected individuals this analysis revealed two distinct groups of individuals (Figure 1A). A majority of the individuals clustered in the low percentages (< 4% maximum PD, < 3% maximum DB), while four exhibited high percentages of both parameters (> 7% maximum PD, > 6% maximum DB). One of these (ZM215F; arrowhead) had been shown previously to be a case of co-infection with two genetically distinct variants from a single donor differing by a PD of more than 9% [42,43], while additional phylogenetic analyses (described below) identified the remaining three individuals (black arrows) as cases of superinfection (ZM282M, ZM211F, and ZM247F).

A representative Highlighter plot http://www.hiv.lanl.gov was compiled for each individual's population sequences and provided a visual representation of homogeneity or heterogeneity of the viral population at each time point relative to the virus population at the time of the acutely infected partner's seroconversion (month 0) (Figure 1C-F). Nucleotide changes from the month 0 sequence are demonstrated with tick marks that bear colors unique to each nucleotide (A = green, T = red, G = orange, C = light blue, Degenerate/ambiguous = dark blue; http://www.hiv.lanl.gov/content/sequence/HIGHLIGHT/help.html).

This analysis showed that for a majority of the acutely infected individuals (18/22), the gp41 sequence remained relatively homogeneous with no evidence of dual infection. An example of this is shown in Figure 1C for subject ZM289M, who exhibits only minimal changes (1 base change at 3 months, 12 months, 24 months, and then 2 changes at 30 months) in the gp41 sequence of the infecting HIV-1 strain over 30 months. However, in the three individuals identified above evidence of superinfection was obtained (Figure 1D-F). In addition, for one individual, ZM215F, evidence for co-infection by distinct variants from a single donor was observed, confirming a previous study [43].

ZM282M (Figure 1D) has few base changes until 10 months, at which point there are 49 degenerate base changes observed, consistent with a mixture of genetically distinct viruses at this time-point. Interestingly, the Highlighter plot shows that the superinfecting virus present at 10 months persists until 36 months when there was evidence for emergence of a dominant recombinant virus (see below). This superinfecting virus predominance is evident from the resolution of mixed bases (blue ticks, representing mixed bases) to simple mismatched bases that are derived from the superinfecting strain compared to the month 0 viral sequence.

Individual ZM247F (Figure 1E) was previously reported to be co-infected by closely related variants (PD 2.7%, corresponding to 11 nucleotide differences in gp41) from the same individual at the time of acute infection [43]. At month 3 post-infection there is evidence of superinfection by a genetically distinct virus (PD ~12%) that at this time point becomes the predominant strain, then at 18 months and 21 months, a significant number of degenerate bases are observed (40 and 36, respectively) consistent with a re-emergence of the initial virus strain that results in a mixture of it and the superinfecting virus in the plasma. At 24 months, a recombinant of the superinfecting strain again starts to dominate.

ZM211F (Figure 1F) resembles ZM282M in that the virus sequence is homogeneous until month 9 where there is clear evidence for superinfection, with a mixture in the viral population as seen by degenerate bases. By month 18, the superinfecting virus sequence has become dominant and remains the predominant strain until at least 33 months.

Thus through a combination of degenerate base and phylogenetic analyses on longitudinal sequenced samples, we identified three cases of superinfection in this cohort. In order to rule out the possibility that we might have missed cases of superinfection because of rapid recombination between the superinfecting virus and the initial infecting variant [44], we performed the same analysis on a 400 bp segment of the *gag *gene encoding a region of p24. The results of this analysis (data not shown) did not reveal any additional cases of superinfection.

#### Clinical characteristics of superinfected individuals

Table 1 shows the sexual behaviour data collected from self-reported questionnaires for the 22 acutely infected individuals abstracted from 2002-2010. The three acutely infected individuals that were superinfected (ZM282M, ZM247F, ZM211F) are compared against the 19 acutely infected individuals that were not superinfected. The comparison between groups was limited to the first 12 months of primary infection during which initial superinfection was observed. The ages were similar between the two groups. Genital infections or ulcers were reported or visualized in all three participants in the superinfected group, and in 36% of the not superinfected group, which was statistically significant (p = 0.01). Two individuals in each group had a positive RPR titer in the first year of their HIV-1 infection (p = 0.02), and there was no difference between *Trichomonas *infection within the couple between the groups (p = 0.31). All individuals in the superinfected group had sexual intercourse with at least one partner between each visit (total of 12 visits over 1 year for 3 individuals). The superinfected group reported 201 episodes of sexual intercourse with condoms and 29 (12.6% of total) episodes of sexual intercourse without condoms (mean values are shown in Table 1). Sex with a non-spousal partner (11 episodes) was reported by one individual, ZM282M, while both superinfected women denied extra-marital contact. The non-superinfected group reported 2042 episodes of sexual intercourse with condoms, and 104 episodes (4.8%) without condoms. Three individuals in the not superinfected group reported having extra-marital partners (ZM249M, ZM250M, ZM184F). Only 1 of the newly infected women became pregnant, although 4 of the cohabiting female partners of the 13 acutely infected men became pregnant. All men in this study were uncircumcised.

**Table 1:** Reported sexual activity of newly infected partner in 12 months post-primary infection

Variable	Not Superinfected (n = 19)	Superinfected (n = 3)	p-value
Age	28	26	0.41

Female gender	47%	66%	0.43

Reported genital ulcer/infection	7/19	3/3	0.01

Sex with partner/no condom, mean (range)^#^	5.5 (0-26)	9.7 (2-24)	0.57

Sex with partner/with condom, mean (range)^#^	107.4 (10-322)	67 (11-112)	0.51

Sex/non-spousal partner (contacts; fraction reporting exposure)	5; (3/19)	11; (1/3)*	0.27

RPR positivity	2/19	2/3	0.02

*Trichomonas *(in female partner)	4/19	1/3	0.31

Pregnancy	1/9	0/2	0.70

* self report - rare for women

^# ^mean value/yr in first year

### Analysis of incidence

The incidence of superinfection was determined over 12 month periods after seroconversion, and this was compared against the calculated incidence of primary transmission within the larger cohort of enrolled sero-discordant couples. The first 12 months showed 22 couples with an incidence of superinfection of 13.6 (5.2-34.8, 95% CL) per 100 person years (py). During months 12-24 and 24-36, there were no further cases of superinfection in the remaining 19 patients.

All person-years of observation in the prospective study were used to calculate overall HIV-1 incidence rates in the broader cohort. Seroconversion and transmission rates were calculated including all seroconversions. Exact distribution methods were used to calculate 95% confidence intervals. In the first 3 months, the rate was 13.1 (10.6-16.1, 95% CL), from 3-12 months after enrollment, the rate was 7.9 (6.5-9.4, 95% CL), and the rate between 12-24 and 24-36 months was 7.4 (5.9-9.0, 95% CL) and 7.2 (5.4-9.3, 95% CL), respectively. The higher incidence of infection observed during the first 3 months following enrollment likely reflects infections acquired immediately prior to couples counseling and condom provision and that were still in the antibody negative phase at the time of enrollment.

A comparison of incidence of superinfection in the recent seroconvertors during the first year (13.6/100 py) to the incidence of primary infection (per 100 person years) in the broader discordant couple cohort during either the 0-3 month (13.1) or 3-12 month (7.9) periods using the t-test assuming equal variance yielded no statistically significant differences.

### Characterization of superinfection by single genome amplification

**Figure 2:**
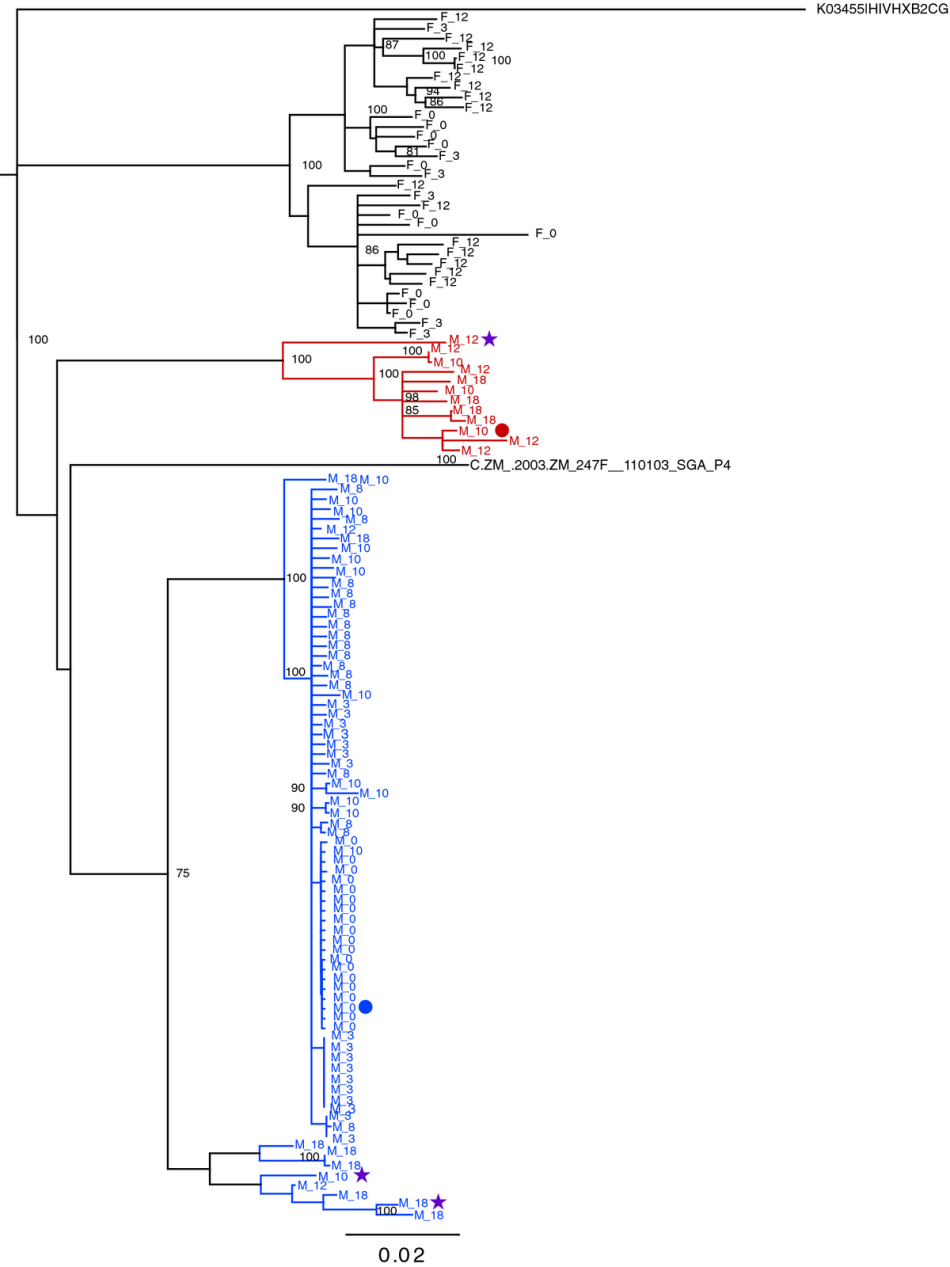
**Neighbor-joining tree of full-length SGA *env *sequences for ZM282M**. Blue and red sequences represent the initial infection and superinfection sequences, respectively, from the acutely infected subject ZM282M; black sequences are derived from the epidemiologically unlinked cohabiting partner ZM282F, who is chronically infected. The time points are indicated along with M or F for each sequence (i.e. M_8 is the acutely infected ZM282M at 8 months post seroconversion). The ZM282F "0" time point corresponds to seroconversion of ZM282M. The duration of infection for ZM282F is not known. Bootstrap values > 80 are considered statistically significant. Sequences denoted by circles indicate the parental sequences (blue, red circle) and those denoted by stars identify potential recombinant daughter sequences that were used for recombination analyses (below).

In order to better understand the dynamics of superinfection in the three individuals identified, single genome amplification (SGA) of full-length *env *gene was performed. The neighbor-joining (N-J) phylogenetic tree of the sequences obtained for the male and female in couple ZM282 is shown in Figure 2. Sequences from the chronically infected partner, subject ZM282F (black), cluster distinctly from the male's sequences (blue), confirming that these individuals are an unlinked transmission pair, and her sequences exhibit up to 3.5% diversity (pairwise distance) consistent with that of a chronically infected individual. For the male, the nearly identical sequences from the earliest time point (M_0) branch together, consistent with a genetic bottleneck in which a single genetic variant was transmitted [42,45,46]. Limited genetic heterogeneity was observed over the next 8 months with the *env *sequences differing by only 1.1% over this time. By contrast, at the M_10 time point, two distinct virus populations were detected, with approximately 1/3 of the sequences forming a distinct, genetically distant branch that is approximately 12.5% divergent from the initial infecting virus (red). This is consistent with the time of superinfection observed from population sequence analysis of the gp41 encoding region (Figure 1D). At subsequent times (12 and 18 month) there are sequences present that cluster with the superinfecting virus and others that form another distinct branch that represent recombinants (see below). Only a minority of the sequences from the later time points cluster with the initial infecting virus population, consistent with the superinfecting virus becoming the dominant viral population, as was also observed in gp41 Highlighter plot for this individual (Figure 1D).

**Figure 3:**
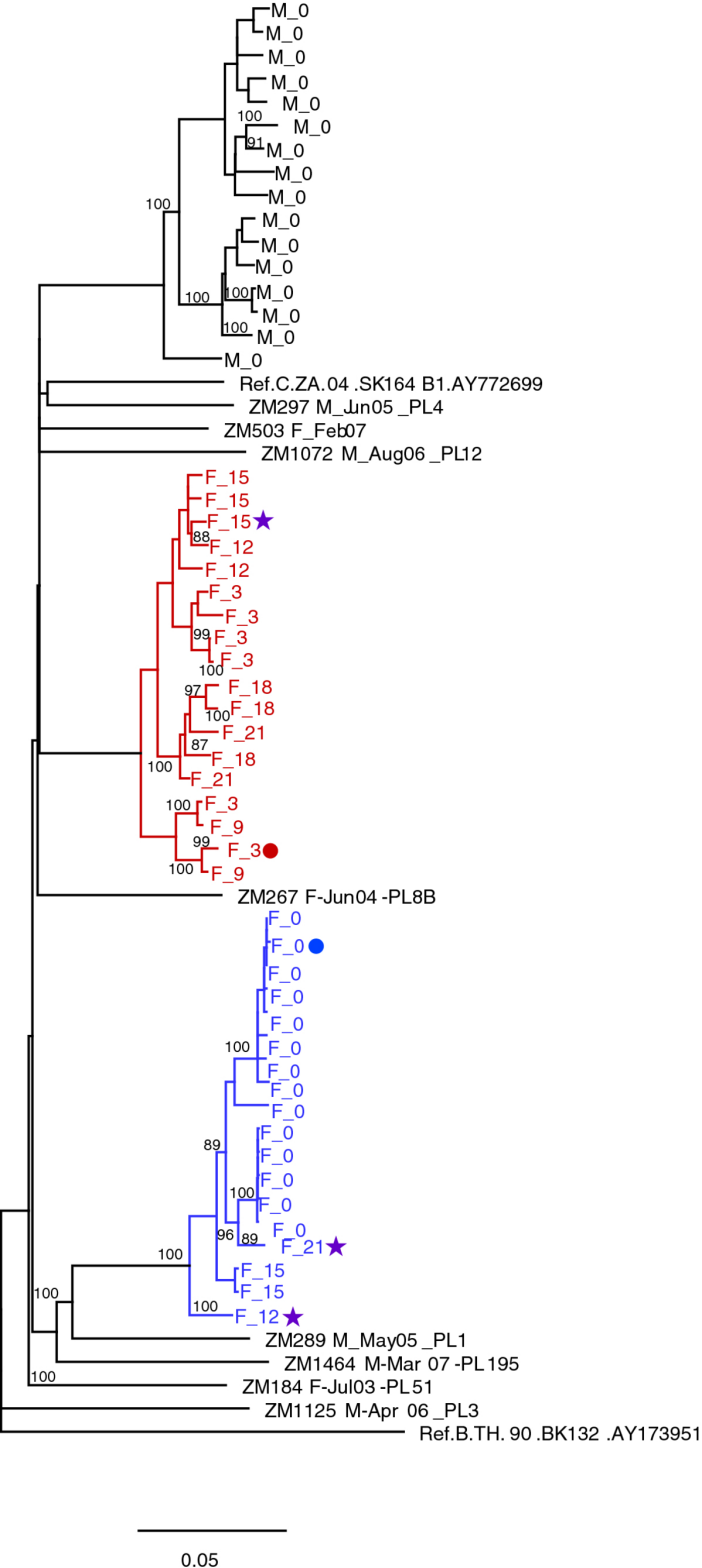
**Neighbor-joining tree representing full-length SGA *env *sequences for ZM247F**. Blue and red sequences represent the env SGA sequences from acutely infected ZM247F (blue) and superinfected (red) viral sequences. Black sequences are derived from ZM247M, the epidemiologically unlinked chronically infected partner. Bootstrap values > 80 are considered statistically significant. Sequences denoted by circles indicate the parental sequences (blue, red circle), and stars denote potential recombinant daughter sequences (purple stars) that were used for recombination analyses (below).

Another example of almost complete dominance by the superinfecting virus is seen with ZM247F. This individual was initially infected by two variants that differ by 2.7% from the same donor, evidenced by two distinct branches of almost identical sequences [43] (Figure 3). Consistent with the Highlighter analysis (Figure 1E), all of the sequences amplified from the three-month time point cluster independently from the initial infecting viruses (red). These later sequences diverge over time and include recombinants with the initial infecting virus (see below), confirming the co-existence and genetic interaction of both the initial and superinfecting virus strains.

**Figure 4:**
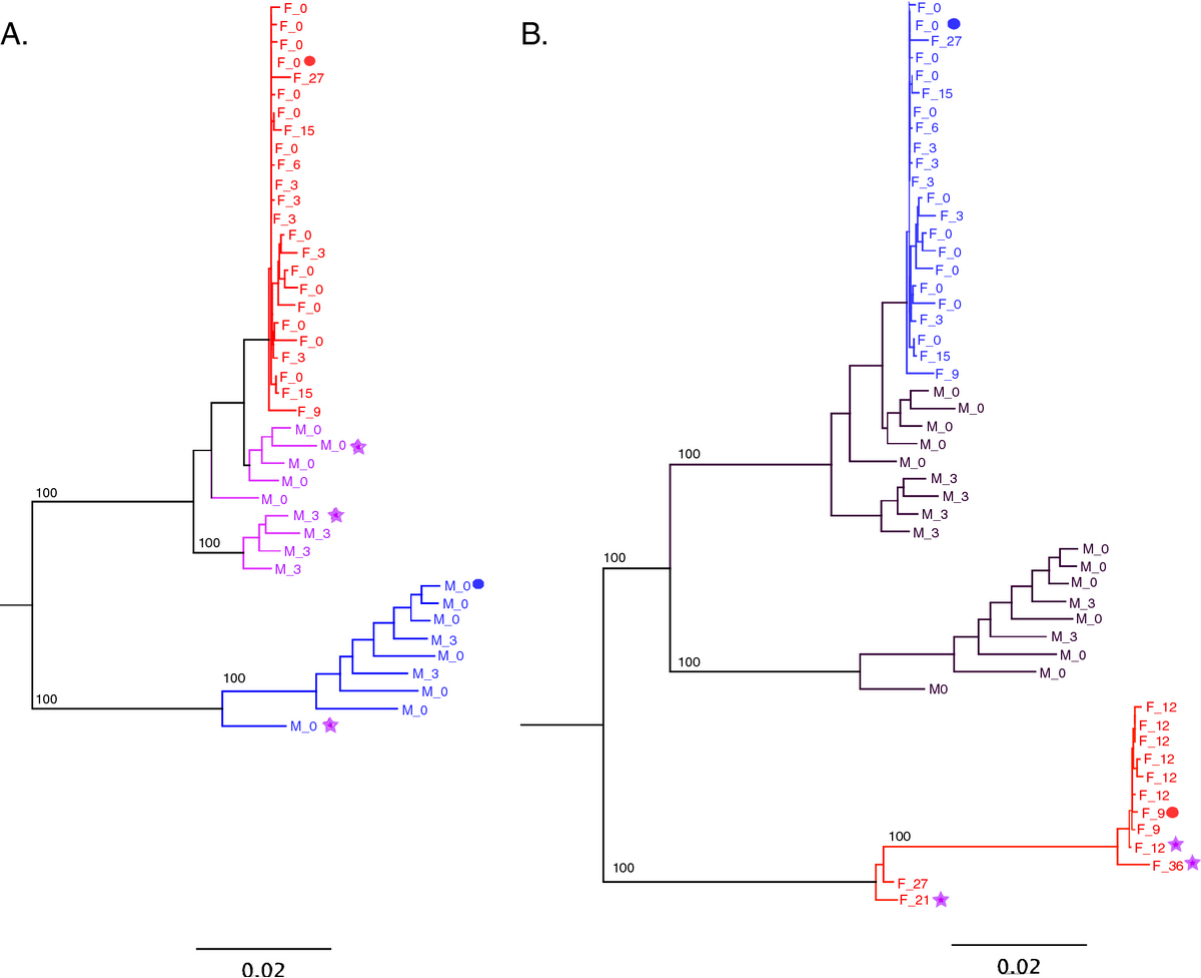
**Neighbor-joining tree of full length SGA *env *sequences for ZM211M and ZM211F**. (**A**) The chronically infected ZM211M sequences are depicted in blue, and the superinfecting ZM211F sequences are depicted in red, while those in purple represent potential recombinant sequences between the blue and red sequences. (**B**) An expanded phylogenetic tree showing time points 0-36 months for the acutely infected ZM211F initial infecting virus (blue) is distinct from ZM211M (black). Sequences denoted by circles indicate the parental sequences (blue, red circle) and stars denote potential recombinant daughter sequences (purple stars) that were used for recombination analyses described below.

Figure 4 illustrates an example of what appears to be superinfection of the chronically infected male partner by his acutely infected spouse (ZM211M, Figure 4A), and superinfection of the newly infected female partner nine months later from an outside source (ZM211F, Figure 4B). In this case the chronically infected partner, ZM211M (Figure 4A), has evidence of a distinct, diverse, cluster of *env *variants, at the time of his partner's seroconversion that represents the chronic viral population (blue). However, in contrast to the chronically infected partner in the other 2 cohabiting couples (ZM282 and ZM247), there is evidence for superinfection in the male at the time of his partner's seroconversion, with a subset of sequences that cluster closely with the woman's acute sequences (purple). Three months later, there is evidence of recombinant variants developing that contain a greater fraction of the man's sequence (see below). We interpret these findings to indicate that during acute infection, the woman partner transmitted her genetically distinct virus to her spouse, who died 6 months later. However, because plasma samples were not available prior to the woman's seroconversion time-point, it is possible that the male was infected by two phylogenetically distinct viruses and that one of these is the source of his spouse's (ZM211F) primary infection. Recombination analyses described below are most consistent, however, with superinfection of the male by his acutely infected partner.

ZM211F, the acutely infected partner, has a homogenous viral population at her earliest time point, which developed very limited diversity over the first 6 months (Figures 1F, and 4B). At the 9-month time point, ZM211F exhibits clear evidence of superinfection by a virus (red) that is genetically distinct from her partner's (black).

**Figure 5:**
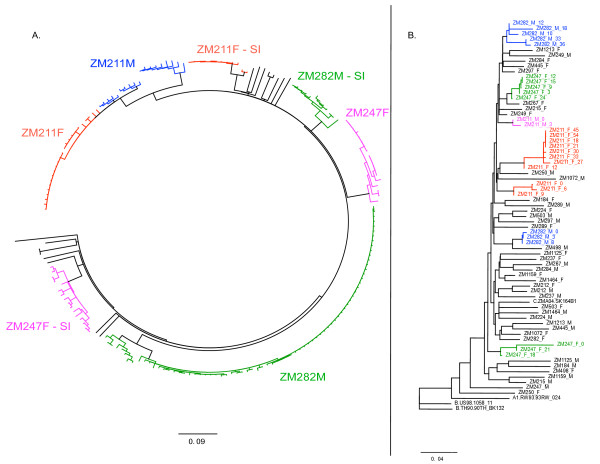
**Neighbor-joining trees of SGA *env *and population gp41 sequences for the cohort**. (**A**) Neighbor-joining phylogenetic tree of single genome amplified *env *gene sequences from each superinfected individual in the context of Zambian sub-type C *env *sequences. The Zambian subtype C sequences include twelve of the non-superinfected, newly infected individuals from this study. Superinfected individuals are assigned discrete colors and the superinfecting quasispecies is denoted by SI. (**B**) Neighbor-joining tree of gp41 sequences for all 22 couples. Superinfected individuals are assigned discrete colors.

In order to rule out evidence that superinfections might have originated from contaminating sequences within the cohort, we analyzed the *env *SGA sequences from the three superinfection pairs in the context of contemporaneous sequences from the cohort (Figure 5A). In each case a distinct superinfecting genetic variant could be identified which segregated independently on the phylogenetic tree. The extra-cohort origin of these superinfecting strains was further supported by a phylogenetic analysis of gp41 population sequences for the viruses from all 22 couples (Figure 5B)

### Recombination analysis using highlighter tool

One consequence of superinfection can be the generation of novel unique recombinant viruses, and evidence for HIV-1 superinfection can be further supported in the 3 couples analyzed here by evaluation of the sequences for recombination. For each superinfected individual, parental viruses were selected by generating a consensus of full-length *env *SGA sequences from the time of seroconversion and choosing a full-length *env *SGA amplicon sequence that matched the consensus sequence (filled blue circle). Similarly, parental superinfecting viruses were selected by comparing the chosen viral *env *sequence against all *env *sequences at the time of superinfection and selecting the superinfecting *env *sequence with the greatest pairwise distance from the seroconversion virus (filled red circle).

**Figure 6:**
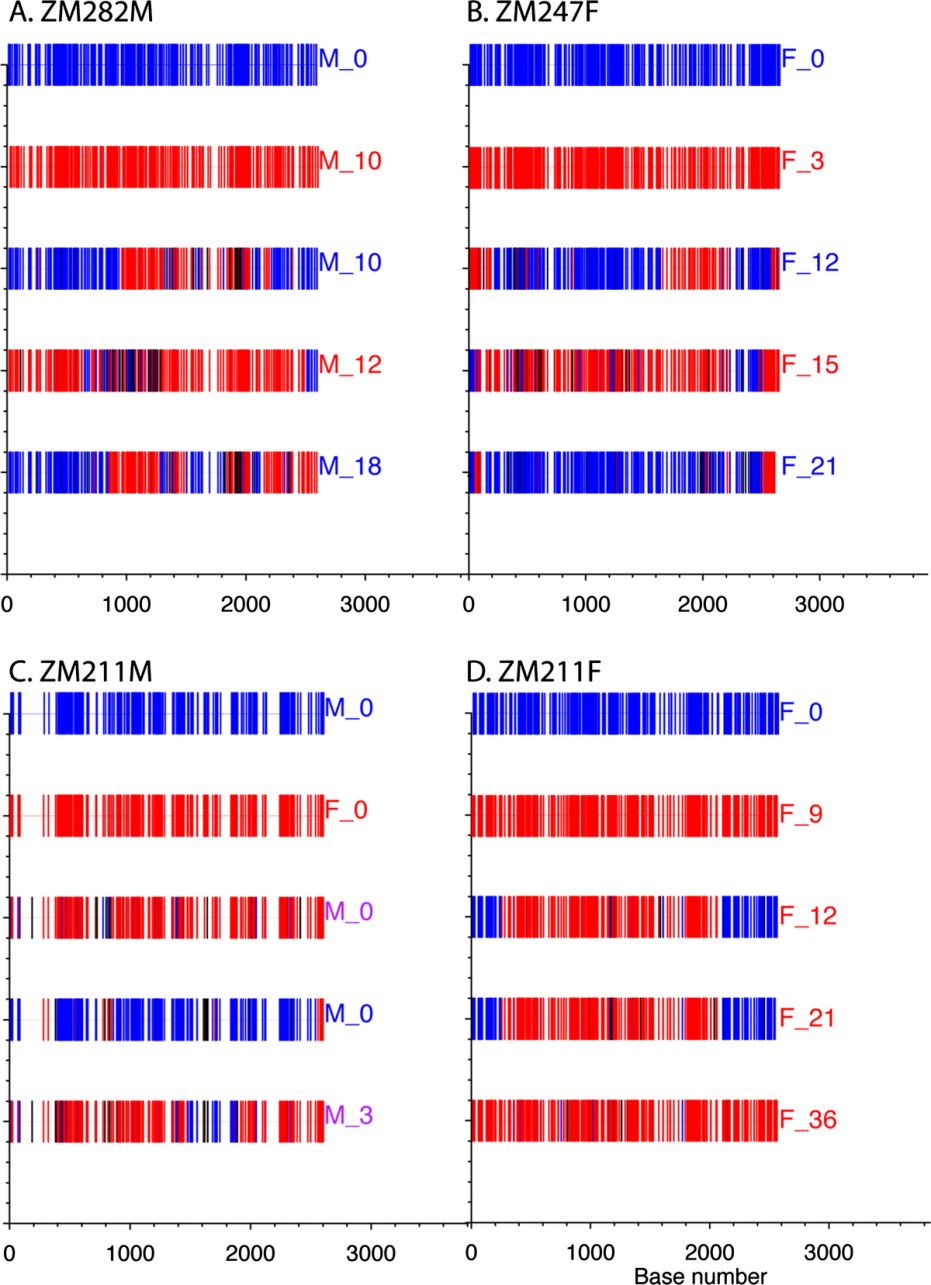
**Highlighter recombination plots of full-length *env *for each individual who was superinfected**. Highlighter recombination analysis of sequences from ZM282M (**A**), ZM247F (**B**), and ZM211M (**C**) and ZM211F (**D**). The parental virus sequence from the acutely infected or a representative parental sequence from the chronically infected partner (ZM211M) are shown in blue in the first bar of each panel. The superinfecting parental sequence shown in red is the second bar. The presumptive recombinant daughter sequences from three representative time points are shown below the parents for each case of superinfection.

Figure 6A clearly shows that for each of the selected ZM282M sequences recombinant variants have been generated (purple stars, Figure 2). In the ZM282M_10 recombinant sequence for example, the C-terminal region of gp120 and N-terminus of gp41 of the initially infecting virus (blue) have been replaced by the superinfecting virus sequences (red). By contrast, as might be predicted from its position on the N-J tree, ZM282M_12 consists almost entirely of superinfecting virus sequences, with only small regions of gp120 and the C-terminus of gp41 originating in the initial acutely infecting virus.

In the case of ZM247F (Figure 6B), however, despite very distinct recombination patterns across the *env *gene in the three cases chosen, a recombination break point within the C-terminal domain of gp41 (residue 2200), first observed at 12 months (F_12), was conserved in the 15 and 21 month sequences even though these variants were located in distinct clusters on the N-J tree. This suggests that this recombination event may have conferred a specific fitness advantage.

In couple ZM211, we compared presumed recombinant viruses in the chronically infected partner ZM211M with virus sequences from both the ZM211M quasi-species (blue circle, Figure 4A) and the ZM211F initially infecting viruses (red circle, Figure 4A). In the latter case (Figure 6C) the recombinant viruses clustering closely to the newly infecting ZM211F founder sequence (purple stars, Figure 4A) exhibited discrete recombination events, but also showed evidence of conserved recombinant regions derived from ZM211M at the very N-terminus of gp120.

Finally, a comparison of three putative recombinants in the superinfecting population of ZM211F (purple stars in Figure 4B) to the initial virus and the superinfecting strain at 9 months (ZM211F_9) revealed clear evidence of recombination in the superinfecting strain with acquisition of *env *sequences from the initially infecting virus at the N-terminus of gp120 and the C-terminal domain of gp41 being evident at 12 and 21 months. Thus in each of the four superinfected individuals, we observed extensive recombination with evidence for co-existence of diverse recombinants at time points subsequent to superinfection.

## Discussion

Understanding the mechanism for HIV-1 superinfection is crucial to the development of an HIV vaccine in order to prevent HIV-1 acquisition in a naïve host, since HIV-1 superinfection calls into question the idea that a robust primary immune response to HIV-1 infection provides some immunological protection from re-infection with a heterologous HIV-1. Superinfections occurring in heterosexual cohabiting pairs have not been studied in detail or longitudinally, despite the fact that most primary infections occur in HIV-1 discordant couples [40,41]. Though discordant couples are considered 'high risk,' they are not typically thought to be exposed to as many different viruses as sex workers or intravenous drug users [31,47]. For this reason, it might be predicted that superinfection would be observed at lower frequency and would occur predominantly between individuals within a couple; however, this was not the case. In the 22 newly infected partners, who had acquired HIV outside the marriage, we observed a frequency of superinfection in these individuals in the first year of follow up that was similar to that of primary infection (13.6% vs. 7.8%, p > 0.05). Even though we excluded couples in which the chronically infected partners had viral loads lower than 1000 from this study, all of the superinfections in the seroconverting partner originated from a non-spousal partner. Thus, it is clear that these couples are a higher risk subset of the cohort with exposure to HIV-1 infection outside the main partnership. The very limited frequency of intra-couple superinfection (1/4) studied here in part reflects continued safe sexual practices within the couple, since greater than 95% of reported sexual activity was with a condom. Nevertheless, significant numbers of non-condom exposures did occur (104/2146) in 19 of the 22 newly infected partners who did not become superinfected. Although self-report of external sexual activity is clearly underreported [37], it seems unlikely that for each of the three superinfected individuals, the number of unprotected extra-marital exposures would exceed the number within the marriage. Moreover, with an adult seroprevalence rate of ~20% in Zambia, extramarital exposures should in a majority of instances be with seronegative individuals and therefore present less of a risk of potential superinfection than with the known seropositive partner. Interestingly, a similar lack of intra-couple superinfection has been observed in a recent study of 11 seroconcordant couples infected with disparate viruses in Uganda [48].

One factor that could influence susceptibility to superinfection is the presence of sexually transmitted diseases. Since genital infections and ulcers break down mucosal barriers and contribute to increased risk for primary HIV infection [42,49,50]. In the analysis of behavioral characteristics and clinical signs, the factors that trended toward significance were the presence of GUD on physical exam and RPR positivity in the superinfected group as compared to the non-superinfected group, although 7/19 non-superinfected individuals did have GUD. Previous studies in this Zambian cohort have shown a 2-3 fold increase in risk of HIV-1 infection in uninfected partners with GUD, after correction for viral load in their chronically infected partner [51-53]. In contrast to this higher-risk group, longitudinal *gag, pol*, and *nef *gene sequence data from 80 epidemiologically linked transmission pairs in the cohort (where transmission was from the cohabiting spousal partner) in the first two years of follow-up have not demonstrated any evidence of superinfection from non-spousal partners (data not shown), consistent with a lower frequency of extra-marital sexual activity in this cohort subset.

Despite the fact that a majority of the acutely infected individuals in this study of ZEHRP transmission pairs have > 2 years of follow-up, HIV-1 superinfection was observed within the first year of follow-up in each of the 3 acutely superinfected individuals. This is consistent with recent studies of intra-subtype superinfection in subtype B infected individuals, where in one case mathematical modeling indicated a 21-fold reduction of superinfection after 1 year of infection [25], and in a second case, a retrospective analysis of individuals in the San Diego and Los Angeles Acute HIV Infection and Early Disease Research Programs demonstrated 3 cases of superinfection within 13 months of seroconversion [17]. In contrast, the timing of superinfection in a subtype A commercial sex worker cohort appears less constrained, with superinfection detected as late as 5 years after primary infection [26].

The analysis of longitudinal *env *sequences, amplified by the SGA approach, for each of the individuals identified through degenerate base analysis allowed the definitive resolution of both the timing and nature of superinfection. In each of the three recent seroconversion cases a distinct superinfecting genetic variant could be identified which segregated independently on the phylogenetic tree (Figure 5A). Recombination between the primary infection variant and the superinfecting variant was observed in each case; and at some time points, consistent with the Highlighter analyses of population sequences, these recombinants became the dominant variant in the circulating virus population. Interestingly, we observed the conservation of recombination break-points within different variants in an individual over several months, suggesting that recombinant viruses with these particular sequence mixes possess fitness benefits over either the initial or the superinfecting strain. This is consistent with the observation of Streeck et al., [36], who showed that recombination between initial and superinfecting viruses could accelerate immunological escape from cellular immune responses. In a more global sense, the selection of mixed genotypes with enhanced population fitness is evidenced by the numerous circulating recombinant forms of HIV-1 resulting from dual infection of individuals [9,33,34], which clearly contribute to the overall diversity of a virus population. Additional studies will be required to fully characterize the basis of recombinant virus selection in the subtype C infected individuals under study here.

The SGA analysis of viral sequences bolstered our interpretation that ZM211M was superinfected from his spousal partner, ZM211F, during her acute seroconversion. At the time of her seroconversion, ZM211M has two dominant and distinct quasispecies with limited evidence for recombination between them. In contrast at month 3, a distinct population of recombinant viruses arises. This is consistent with superinfection of ZM211M during his spouse's acute viremia (viral load greater than 750,000), followed by the emergence of recombinants. Moreover, shortly after the probable superinfection, the viral load of ZM211M increased 10-fold and he is deceased within 6 months.

Determining why HIV-1 does or does not superinfect an exposed individual will be crucial to understanding the nature of an immune response that is capable of preventing *de novo *infection. Given the considerable antigenic dissimilarities between subtypes, we might not expect that initial infection by one subtype of HIV-1 would provide significant immune protection against other subtypes; on the other hand we might expect there to be some protection from reinfection of infected patients by more closely related HIV-1 strains of the same subtype. This does not appear to be the case during the first year of infection in the subtype C infected individuals studied here, where rates of intra-subtype superinfection in the first year of study were similar to those of primary infection [39]. However, it is of interest that in the three individuals that are superinfected, little variation in *env *sequences is observed in the period prior to the superinfection event, suggesting that there may be limited neutralizing antibody pressure on the founder virus. Indeed, preliminary studies indicate the absence of potent neutralizing antibody responses to the founder virus at the visit prior to superinfection (D. Basu et al., unpublished). It will be of interest to determine whether there is a more potent neutralizing antibody response in the non-superinfected individuals who also report extra-marital contact. Moreover, given that in this study each partner in the couple is infected with a different strain of subtype C HIV-1, it is possible that repeated exposure to a partner's HIV-1 strain could stimulate the development of HIV-1 specific immune responses and that this might have provided protection against intra-couple superinfection. This type of immune stimulation with boosting of the cellular immune response has been reported to occur in subtype B infected men who have sex with men [23].

The existence of HIV-1 superinfection presents an obstacle to develop a vaccine to prevent primary infection with HIV-1. With technologies such as next-generation sequencing being employed to detect HIV-1 superinfection [48], the detection of very small viral sub-populations at a given time point will increase resolution. There are behavioral and clinical aspects (e.g. circumcision, genital ulcers) that influence this phenomenon but there are likely immunologic correlates that render some individuals more susceptible to superinfection. Continued study of HIV-1 superinfection within cohabiting heterosexual couples can provide insights into such correlates in the context of a potentially highly susceptible and relatively low-risk cohort type.

## Methods

### Zambian cohort

The Zambia Emory HIV Research Project (ZEHRP), a Rwanda Zambia HIV Research Group (RZHRG) site in Lusaka, Zambia, was established in order to study heterosexual cohabiting HIV-1 discordant couples, and provides voluntary testing and counseling as well as long-term monitoring and health care to participating couples [54,55]. HIV discordant couple is defined in this cohort as a couple that upon screening and enrollment has one HIV-infected partner (seropositive index partner) and one HIV-uninfected partner [56]. This screening is based on rapid HIV-1 antibody test positivity [54,57]. Both partners are followed quarterly with repeat counseling and documentation of reported sexual exposures within and outside the marriage, and assessment of biological markers of unprotected sex [37]. Plasma from the seronegative partner is tested at every visit for HIV-1 antibodies with rapid tests, and for the presence of p24 antigen using the Vironostika^® ^HIV-1 p24 antigen ELISA [54,57]. Despite counseling and provision of condoms, and a two-thirds reduction in transmission [58], approximately 7%-8% per year of the initially seronegative partners are infected by HIV-1. Once a transmission event had been established, the newly infected partner was followed quarterly, and the chronically infected partner at least annually. Blood products (PBMC and plasma) were collected at each visit under protocols approved by the University of Zambia Research Ethics Committee and the Emory Institutional Review Board. Plasma was obtained by centrifugation of whole blood, and stored in aliquots at -80°C until use. Viral RNA was extracted from these samples using the QIAamp^® ^Viral RNA Mini kit (Qiagen Inc., Valencia, CA). Individuals who meet criteria for antiretroviral therapy are referred elsewhere and drop out of the cohort studies.

Viral gp41 sequences from newly infected, previously seronegative, individuals and their chronically infected partners were used to define epidemiologic linkage of the transmission as described by Trask et al., 2001 [41]. During the period 01/01/2002 to 06/01/2007, a total of 202 seroconversions were identified, of which 49 (24%) were classified as unlinked. A total of 22 couples were selected for further study based on the criteria: 1. Samples corresponding to at least one year of follow-up were available at Emory University, and 2. The seropositive index partner had a viral load greater than 1000, because we were interested in determining the frequency of superinfection within the couple and primary transmission from such individuals is rare. A table detailing the available demographic, clinical and behavioral characteristics of these individuals is provided in Additional file 1: Table S1. Although the exact time of transmission is not available, for each newly infected partner, samples were collected within a median of 91 days (range 10-181 days) of the last seronegative visit. For this study, we have defined two different types of dual infection: co-infection is defined as the detection of two genetically distinct viruses at the time of seroconversion in the previously HIV-1 negative partner; superinfection is defined as the detection of more than one genetically distinct virus at least 3 months after primary infection seroconversion in the seronegative partner. For the seropositive index partner, superinfection was defined as the detection of a novel genetically distinct variant at or after the time of infection of their seronegative partner.

### HIV-1 gp41 and *gag* nested PCR

Viral RNA was reverse-transcribed using SuperScript^® ^III One-Step RT-PCR System with Platinum^® ^Taq High Fidelity as per manufacturer's guidelines (Invitrogen Co., Carlsbad, CA). Nested PCR amplifications using Expand High Fidelity polymerase (Roche Applied Science, Indianapolis, IN) were performed for gp41 as previously described and in Additional file 2: Methods [41]. Purified positive amplicons were sent out for direct sequencing to MWG Sequencing (Huntsville, AL). Nested PCR for *gag* was accomplished using the following primers: Outer: 5' - TTC TAC GGA GAC TCC ATG ACC C - 3', 5' - ATT TGA CTA GCG GAG GCT AGA A - 3', Inner: 5' - ATT GCT TCA GCC AAA ACT CTT GC - 3', 5' - CGA CCA AAA TTA CCC TAT AGT GCA G - 3', and sequencing primers: 5' - GGG ACA TCA AGC AGC CAT- 3', 5' - GCC AAA GAG TGA TTT GAG GG - 3'.

### Sequence analysis and highlighter analysis

Sequences were analyzed from amplicons in Sequencher 4.8 (Gene Codes Corporation, Ann Arbor, MI). Geneious Pro (Biomatters Ltd, Auckland, New Zealand) software was used to align sequences, and neighbor-joining trees were generated using the Tamura-Nei genetic distance model with the bootstrap resampling method. Additionally, the Highlighter tool from Los Alamos National Laboratory HIV Sequence Database http://www.hiv.lanl.gov/content/sequence/HIGHLIGHT/highlighter_top.html was used to map mutations deviating from the earliest sample. APOBEC G to A mutations (open diamonds) and degenerate bases (Dark Blue) were quantified in a longitudinal fashion within the acute transmission partner's virus with respect to the viral sequence from the time of seroconversion. Recombination analysis was performed using the Highlighter tool for analysis of the presumed parent and daughter sequences.

### Env single genome analysis (SGA)

Single genome PCR amplification was performed of the entire *env *gene [42,43]. Single genome analysis was conducted on couples who were determined to have dual infection by the screening methods of degenerate base counting, HMA or phylogenetic analysis of sequences encoding gp41. Full-length *env *gene sequences were analyzed for superinfection cases (ZM211M, ZM211F, ZM282M, ZM247F).

### Degenerate base counting

After obtaining the sequences from gp41 PCR amplicons, degenerate or ambiguous codes were manually counted using the International Union of Pure and Applied Chemistry (IUPAC) designations. Only nucleotide positions where the secondary (and occasionally tertiary) peak was at least 30% as high as the primary peak was counted as a mixed peak, and these had to be present in both forward and reverse primer sequences. Degenerate codes were then assigned to the mixed nucleotide accordingly.

### Heteroduplex mobility assay

Second round gp41 PCR products amplified from plasma were used directly in the heteroduplex assay as described elsewhere [8,59].

### HIV-1 quantitative viral loads

HIV-1 viral load determination was performed on plasma using the Amplicor HIV-1 Monitor Test, v 1.5 (Roche Diagnostics, Indianapolis IN).

### Statistical analysis of behavioral data

A univariate analysis to compare the superinfected and non-superinfected groups was performed using the Wilcoxon rank sum test, or Fisher's exact test as appropriate, for continuous variables. Categorical variables were compared using the chi-square statistic. All analyses were performed using SAS^® ^version 9.2 (Cary, NC), and *p*-values < 0.05 were considered to be statistically significant. The 95% confidence interval for incidence of infection was calculated based on the method by Clopper and Pearson [60].

## Competing interests

The authors declare that they have no competing interests.

## Authors' contributions

CSK and DB performed the experiments, analyzed the data and drafted the manuscript. PAH performed the experiments on ZM211F. PTH did the analysis of the pairwise distance for all individuals. EC, JM, and WK are vitally involved in the sample collection in the discordant couple cohort and critically reviewed the manuscript. CD participated in the design of the experiments and critically reviewed the manuscript. OM utilized the HMA for screening for superinfection, directed the sample collection and critically reviewed the manuscript. NHK performed statistical analyses. EH conceived of the study, participated in its design and coordination and critically reviewed the manuscript. All authors read and approved the final manuscript.

## Supplementary Material

Additional file 1 **Table S1**. Click here for file

Additional file 2 **Methods**. Nested PCR reagents and cycling conditions. Click here for file
