# Supplementary material for: Timing and source of subtype-C HIV-1 superinfection in the newly infected partner of Zambian couples with disparate viruses
Source: Retrovirology. 2012 Mar 20;9:22. doi: 10.1186/1742-4690-9-22 (PMC3349552; doi:10.1186/1742-4690-9-22)
Supplement: Additional file 1 — Table S1. [file 1742-4690-9-22-S1.PDF]

Supplementary Table 1

Couple ID	Sex	Age[1]	Acute VL[2]	Index VL	Sub-type	GUD[3]	STI[3]	Trich	RPR	Circum-cision	Preg	Sex with condom[4]	Sex w/o condom[4]	Other partner[5]
ZM184	F	30	71,290	147,239	C	Yes	Yes	Yes	No	No	No	39	9	Yes
**ZM211**	**F**	**30**	**2,640**	**96,689**	**C**	**No**	**Yes**	**No**	**No**	**No**	**No**	**11**	**3**	**No**
ZM212	F	37	10,548	750,000	C	No	No	No	No	No	No	322	5	No
ZM215	F	24	241,190	ND	C	No	No	No	No	No	No	155	26	No
ZM224	F	22	624,803	17,252	C	Yes	Yes	No	No	No	No	24	0	No
ZM237	M	43	44,870	1,323	C	Yes	Yes	No	No	No	NA	41	4	No
**ZM247**	**F**	**23**	**750,000**	**53,854**	**C**	**No**	**Yes**	**Yes**	**Yes**	**No**	**No**	**112**	**26**	**No**
ZM249	M	30	750,000	ND	C	Yes	No	No	No	No	NA	145	25	Yes
ZM250	M	22	65,260	46,215	C	No	No	No	Yes	No	NA	133	0	Yes
ZM267	F	35	750,000	180,355	C	No	Yes	No	No	No	Yes	188	8	No
**ZM282**	**M**	**25**	**750,000**	**56,700**	**C**	**Yes**	**Yes**	**No**	**Yes**	**No**	**NA**	**78**	**2**	**Yes**
ZM284	M	36	750,000	91,583	C	No	No	No	No	No	NA	97	1	No
ZM289	M	27	399,737	6,692	C	No	No	Yes	No	No	NA	75	8	No
ZM297	M	50	8,687	ND	C	No	No	No	No	No	NA	108	3	No
ZM445	M	32	11,660	22,710	C	No	No	No	No	No	NA	69	4	No
ZM498	F	41	14,220	ND	C	No	Yes	Yes	No	No	No	52	0	No
ZM503	F	22	25,800	ND	C	No	No	No	No	No	No	65	0	No
ZM1072	M	28	36,200	400	C	No	No	No	No	No	NA	144	0	No
ZM1125	M	31	2,006	2,280	C	No	No	No	No	No	NA	153	10	No
ZM1159	M	29	110,220	493	C	No	No	Yes	No	No	NA	90	1	No
ZM1213	M	30	76,286	68,535	C	Yes	No	No	Yes	No	NA	74	5	No
ZM1464	M	29	44,500	34,100	C	No	No	No	No	No	NA	68	0	No

Bolded individuals were confirmed to be superinfected

[1]Age at enrollment

[2]Viral Loads were collected for first antibody positive dates

[3]Genital ulcer disease or STI in 12 months

[4]Sexual data is cumulative events for 12 months

[5]Other partner in the last 12 months

ND=not done, NA – Not applicable
