# Supplementary material for: Timing and source of subtype-C HIV-1 superinfection in the newly infected partner of Zambian couples with disparate viruses
Source: Retrovirology. 2012 Mar 20;9:22. doi: 10.1186/1742-4690-9-22 (PMC3349552; doi:10.1186/1742-4690-9-22)
Supplement: Additional file 2 — Methods. Nested PCR reagents and cycling conditions. [file 1742-4690-9-22-S2.PDF]

NESTED PCR REAGENTS AND CYCLING CONDITIONS:

Enzymes:

Reverse Transcription in One-Step RT-PCR (for population amplification):
SuperScript III One-Step RT-PCR System with Platinum Taq High Fidelity (Invitrogen Co., Carlsbad, CA)

Reverse Transcription for cDNA synthesis (for single genome amplification):
SuperScript III Reverse Transcriptase (Invitrogen Co., Carlsbad, CA)

Nested PCR Amplification:
Expand High Fidelity polymerase (Roche Applied Science, Indianapolis, IN)

gp41 PCR primers and cycling conditions:

Outer Primers:
gp41F1: 5'- TCT TAG GAG CAG CAG GAA GCA CTA TGG G- 3'
gp41R1: 5'- AAC GAC AAA GGT GAG TAT CCC TGC CTA A-3'
gp41 First Round Program:

Step	Temp (°C)	Time/Step (hour:min:sec)
1	50	15:00
2	95	2:00
3	95	0:15
4	50	1:00
5	72	1:00
6		Go to 3, 34 times
7	72	10:00
8	4	forever

Inner Primers:
gp41F2: 5'- ACAATTATTGTCTGGTATAGTGCAACAGCA-3'
gp41R2: 5'- TTAAACCTATCAAGCCTCCTACTATCATTA-3'
gp41 Second Round Program:

Step	Temp (°C)	Time/Step (hour:min:sec)
1	95	2:00
2	95	0:15
3	50	1:00
4	72	1:00
5		Go to 3, 34 times
6	72	10:00
7	4	forever

p24 gag PCR primers and cycling conditions:

Outer Primers:
GOF (For): 5'- ATT TGA CTA GCG GAG GCT AGA A-3'
VifOR (Rev): 5'- TTC TAC GGA GAC TCC ATG ACC C-3'
p24 gag First Round Program:

Step	Temp (°C)	Time/Step (hour:min:sec)
1	50	1:00:00
2	94	2:00
3	94	0:15
4	59	0:30
5	68	5:00
6		Go to 3, 10 times
7	94	0:15
8	59	0:30
9	68	5:00 + 5sec/cycle
10		Go to 7, 40 times
11	68	12:00
12	4	forever

Inner Primers:
GIF (For): 5'- TTT GAC TAG CGG AGG CTA GAA GGA-3'
VifIR (Rev): 5'- TCC TCT AAT GGG ATG TGT ACT TCT GAA C-3'
p24 gag Second Round Program:

Step	Temp (°C)	Time/Step (hour:min:sec)
1	94	2:00
2	94	0:15
3	63	0:30
4	68	5:00
5		Go to 2, 10 times
6	94	0:15
7	63	0:30
8	68	5:00 + 5 sec/cycle
9		Go to 6, 24 times
10	68	10:00
11	4	forever

Alternate Inner Primers:
G60 (For): 5'- CGA CCA AAA TTA CCC TAT AGT GCA G-3'
G25 (Rev): 5'- ATT GCT TCA GCC AAA ACT CTT GC-3'
p24 gag Second Round Program:

Step	Temp (°C)	Time/Step (hour:min:sec)
1	95	3:00
2	95	0:10
3	55	0:30

4	72	1:00
5		Go to 2, 34 times
6	72	7:00
7	4	forever

Full-length env primers and cycling conditions:

Outer Primers:

Vif1 (For): 5'- GGG TTT ATT ACA GGG ACA GCA GAG-3'

Ofm19 (Rev): 5'- GCA CTC AAG GCA AGC TTT ATT GAG GCT TA-3'

Env First Round Program:

Step	Temp (°C)	Time/Step (hour:min:sec)
1	95	2:00
2	95	0:15
3	54	1:00
4	68	4:00
5		Go to 2, 10 times
6	95	0:15
7	54	1:00
8	68	4:00 + 5sec/cycle
9		Go to 6, 25 times
10	72	10:00
11	4	forever

Inner Primers:

EA1 (For): 5'- CCT TAG GCA TCT CCT ATG GCA GGA AGA AGC-3'

EN1 (Rev): 5'- TTG CCA ATC AGG GAA GTA GCC TTG TGT-3'

Env Second Round Program:

Step	Temp (°C)	Time/Step (hour:min:sec)
1	95	2:00
2	95	0:30
3	55	1:00
4	72	3:00
5		Go to 2, 29 times
6	72	10:00
7	4	forever

PCR products (5 μl) were evaluated by 1% gel electrophoresis and Qiagen QIAquick PCR Product Purification kits were used to purify positive reactions. Purified positive amplicons were sent out for direct sequencing through Eurofins MWG Sequencing (Huntington, AL).

SEQUENCING PRIMERS:

gp41 primers:
gp41F2: 5'-ACA ATT ATT GTC TGG TAT AGT GCA ACA GCA-3'
gp41R2: 5'-TTA AAC CTA TCA AGC CTC CTA CTA TCA TTA-3'

p24 gag primers:
GagF2: 5' - GGG ACA TCA AGC AGC CAT- 3'
Gag R2: 5' - GCC AAA GAG TGA TTT GAG GG - 3'
G25 (Rev): 5'- ATT GCT TCA GCC AAA ACT CTT GC-3'

Env primers:
For13: 5'- GAG AAA GAG CAG AAG ACA GTG G- 3'
For14: 5'- TAT GGG ACC AAA GCC TAA AGC CAT GTG -3'
For15: 5'- CAGCACAGTACAATGTACACATGGAA
For16: 5'- TTT AAT TGT GGA GGA GAA TTT TTC TA- 3'
For17: 5'- AGC AGC AGG AAG CAC TAT GGG CGC- 3'
For18: 5'- CAT ATC AAA TTG GCT GTG GTA TAT- 3'
Rev14: 5'- ACC ATG TTA TTT TTC CAC ATG TTA AA- 3'
Rev15: 5'- CTG CCA TTT AAC AGC AGT TGA GTT GA- 3'
Rev16: 5'- ATG GGA GGG GCA TAC ATT GCT- 3'
Rev17: 5'- CCT GGA GCT GTT TAA TGC CCC AGA C- 3'
Rev18: 5'- GGT GAG TAT CCC TGC CTA ACT CTA T- 3'
Rev19: 5'- ACT TTT TGA CCA CTT GCC ACC CAT- 3'
